# The Use of Genetic Information to Define Idiopathic Pulmonary Fibrosis in UK Biobank

**DOI:** 10.1016/j.chest.2022.07.027

**Published:** 2022-08-25

**Authors:** Olivia C. Leavy, Richard J. Allen, Luke M. Kraven, Ann D. Morgan, Martin D. Tobin, Jennifer K. Quint, R. Gisli Jenkins, Louise V. Wain

**Affiliations:** a Department of Health Sciences, University of Leicester, Leicester, England; b National Heart and Lung Institute, Imperial College London, London, England; c National Institute for Health Research, Leicester Respiratory Biomedical Research Centre, Glenfield Hospital, Leicester, England

To the Editor:

Idiopathic pulmonary fibrosis (IPF) is a rare disease with prevalence of 50 in 100,000 cases in the UK.^1^

Genome-wide association studies have identified 20 independent single nucleotide polymorphisms (SNPs) that are associated with IPF risk to date.^2^, ^3^, ^4^ A single common SNP in the *MUC5B* gene promoter region (rs35705950) has a large effect on IPF risk with each copy of the T allele that is associated with a 4- to 5-fold increased risk of IPF.^4^^,^^5^

Most datasets for genetic studies of IPF were derived from dedicated IPF cohort studies, registries, and clinical trials, which are usually modest in size. Large general population cohorts, such as UK Biobank, represent a valuable resource for increasing IPF case sample sizes for molecular epidemiologic studies. However, observed effect size estimates for rs35705950 on IPF risk in general population cohorts, for which cases are defined with the use of the International Classification of Diseases, revision 10 (ICD-10)^6^ J84.1 code, are smaller than those that are estimated in clinically-derived datasets.^7^ Although this attenuation could be explained by misclassification of IPF cases, the misclassification may be mitigated by the substantial gain in statistical power that can be leveraged from very large biobanks. However, more accurate classification of cases and control subjects in biobanks could provide more accurate effect estimates for use in further analyses.

Given this, we proposed that the IPF risk effect size of rs35705950 could be used to evaluate and refine the choice of codes to define IPF cases. We applied this approach in UK Biobank.

## Methods

UK Biobank is a prospective cohort study that contains > 500,000 volunteers who were recruited in the United Kingdom from 2006 to 2010 at ages 49 to 69 years.^8^

ICD-10 code J84.1 (“Other interstitial pulmonary diseases with fibrosis”) was used to define IPF from hospital episodes statistics (HES) (2020 release; last admission date: June 30, 2020) and death (May 2020 version; last date of death: May 22, 2020) data, which were available for all UK Biobank participants. Two self-reported pulmonary fibrosis variables were available. At baseline, participants were asked by a trained nurse to self-report any noncancer illnesses (field id 20002), which included “pulmonary fibrosis.” During an online follow-up survey about work environment conducted in 2015, 121,270 participants were asked whether a doctor had ever diagnosed them with IPF (field id: 22135, version July 2017). Primary care data were available for 230,105 participants (last event recorded: August 18, 2019). Eight primary care codes (Read 2 and Read 3) were used to define IPF.^9^

Control subjects were defined as individuals who had linked primary care data that had not been defined as an IPF case in any of the data sources. We further selected control subjects to be similar to cases for age sex, ever-smoker status. Cases and control subjects were all of genetically determined European ancestry.^10^

Association of rs35705950 with IPF risk was tested with the use of logistic regression that was adjusted for the first ten genetic principal components. We compared the effect size (OR) of the association using each IPF definition with that reported by the largest genome-wide association study with the use of clinically defined IPF cases^4^ and a meta-analysis of published rs35705950 studies.^5^ We considered these previously reported rs35705950 IPF susceptibility effect sizes as the “gold standard” against which to evaluate codes for IPF in UK Biobank.

Using only the ICD-10 (HES and death) defined dataset, we evaluated the effect of excluding participants with cooccurring ICD-10 codes (in HES or death) that might indicate misclassification. We then repeated the association by testing for the *MUC5B* SNP and compared the effect size to the gold standard. Specifically, we excluded (1) secondary or other causes of pulmonary fibrosis (previously collated by Bellou et al^11^) (non-IPF pulmonary fibrosis), and (2) J84.1 ICD-10 code occurrence before the year that the most recent clinical guidelines for diagnosis of IPF^12^ that were published in 2018.

## Results

**Figure 1:**
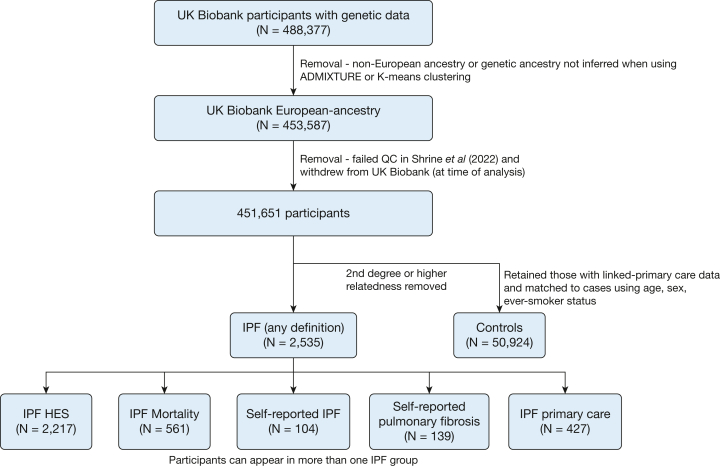
Participant consort diagram. ADMIXTURE and K-means clustering are methods that can be used to infer genetic ancestry. HES = hospital episodes statistics; IPF = idiopathic pulmonary fibrosis; QC = quality control.

**Figure 2:**
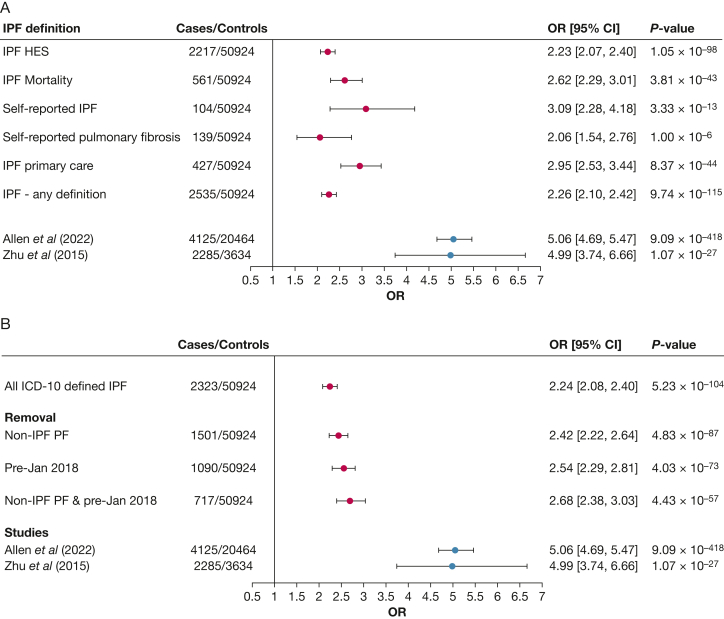
A and B, Effect size estimates of rs35705950 T allele association with IPF risk. Each line shows the effect size estimate and CI for the association between rs35705950 and idiopathic pulmonary fibrosis risk with the use of the different methods for defining idiopathic pulmonary fibrosis in UK Biobank. Estimates in grey are the reference effect size estimates taken from Allen et al^4^(2022) and Zhu et al^5^(2015). A, The use of different idiopathic pulmonary fibrosis case definitions in UK Biobank. Hospital episodes statistics and idiopathic pulmonary fibrosis death defined by J84.1 ICD-10 code. Primary care idiopathic pulmonary fibrosis defined by the following Read 2/Read 3 codes: H563./XE0Yb, H563./X102v, H563./XE0Yb, H563./XE0Yb, H5631/H5631, H5633/X102v, H563z/H563z, H5632/X102u (8). Self-reported idiopathic pulmonary fibrosis defined by UK Biobank field 22135. Self-reported pulmonary fibrosis defined by UK Biobank field 20002. B, With the use of International Classification of Diseases-10 codes and after exclusion of cases with a cooccurring code indicative of being non-idiopathic pulmonary fibrosis pulmonary fibrosis or removing cases defined by the occurrence of a J84.1 code before January 2018. All International Classification of Diseases-10 defined idiopathic pulmonary fibrosis (cases defined using hospital episodes statistics and mortality data only). Non-idiopathic pulmonary fibrosis pulmonary fibrosis code list defined by Bellou et al.^11^ HES = hospital episodes statistics; IPF = idiopathic pulmonary fibrosis.

Of 453,587 European-ancestry participants in UK Biobank, there were 2,535 individuals with one or more codes indicative of IPF; 50,924 individuals were selected as control subjects (Fig 1). SNP rs35705950 was genome-wide significantly associated with IPF risk (*P* < 5 × 10^-8^) for all but self-reported pulmonary fibrosis (*P* = 1.00 × 10^-6^) (Fig 2A). For all definitions, the observed ORs were lower than those previously reported.^4^^,^^5^ Self-reported IPF cases gave an OR closest to previously published estimates. Defining IPF with the use of the J84.1 ICD-10 code in HES data or the self-reported pulmonary fibrosis gave the OR furthest away from previously reported estimates.

Removal of the cases with a cooccurring code that is suggestive of non-IPF pulmonary fibrosis or removal of the cases that are defined by the occurrence of a J84.1 code before January 2018 led to slightly closer effect estimates to those previously reported, but with substantially reduced sample sizes (Fig 2B).

## Discussion

We used association of rs35705950 with IPF risk to evaluate code-based definitions of IPF in UK Biobank. We show that none of the available IPF code definitions, either individually or in combination, replicate the association effect size that is obtained with the use of clinically defined IPF cohorts. We observed that self-reported IPF in UK Biobank provided an effect estimate closest to those previously reported.

We hypothesized that applying code-based exclusions to reduce misclassification among the cases would improve the effect estimates. Although this led to some increase in the effect sizes, they were still < 95% CI of the estimates from IPF studies that used tertiary care diagnoses to recruit participants. Excluding J84.1 ICD-10 code entries that occurred prior to January 2018 was more effective at increasing the OR on its own than removing cases with cooccurring medical conditions that can cause pulmonary fibrosis.

The combined definitions of IPF in UK Biobank gave a prevalence of 559 of 100,000 cases, which is 10-fold higher than population estimates. Because IPF is a rare disease and we used a large control sample, the effect estimate attenuation that we observed for rs35705950 suggests that there is over-estimation of cases in UK Biobank because of low specificity of the definitions that are used.

In conclusion, large biobanks offer an excellent resource for the study of less prevalent common diseases. However, we show that commonly used codes fail to define an IPF case sample that is able to replicate previously reported association effect sizes. Furthermore, pragmatic attempts to refine the phenotype with the use of further code exclusions were unable to improve the estimates. Researchers who use biobanks to study IPF should take these findings into consideration when designing future studies.

## Funding/Support

L. V. W. holds a GSK/Asthma+Lung UK Chair in Respiratory Research (C17-1). L. V. W. and R. G. J. are supported by MRC Programme grant MR/V00235X/1. R. J. A. is an Action for Pulmonary Fibrosis Research Fellow. R. G. J. is supported by a National Institute for Health Research (NIHR) Research Professorship (NIHR reference RP-2017-08-ST2-014). L. M. K. was funded by a Medical Research Council (MRC) PhD studentship (MR/N013913/1). M. D. T. is supported by a Wellcome Trust Investigator Award (WT202849/Z/16/Z). A CC BY or equivalent license is applied to the Author Accepted Manuscript arising from this submission, in accordance with the grant’s open access conditions. The research was partially supported by the NIHR Leicester Biomedical Research Centre; This research has been conducted using the UK Biobank Resource under application 77050. This research used the SPECTRE High Performance Computing Facility at the University of Leicester.

## Financial/Nonfinancial Disclosures

The authors have reported to *CHEST* the following: L. V. W. reports current and recent research funding from GSK, Genentech, and Orion Pharma and consultancy for Galapagos. R. G. J. is a trustee of Action for Pulmonary Fibrosis and reports personal fees from Astra Zeneca, Biogen, Boehringer Ingelheim, Bristol Myers Squibb, Chiesi, Daewoong, Galapagos, Galecto, GlaxoSmithKline, Heptares, NuMedii, PatientMPower, Pliant, Promedior, Redx, Resolution Therapeutics, Roche, Veracyte, and Vicore. J. K. Q. has received grants from The Health Foundation, MRC, GSK, Bayer, BI, AUK-BLF, HDR UK, Chiesi. and AZ and personal fees for advisory board participation or speaking fees from GlaxoSmithKline, Boehringer Ingelheim, AstraZeneca, Chiesi, Insmed and Bayer. None declared (O. C. L., R. J. A., L. M. K, A. D. M., M. D. T.).
